# Prevalence and complication of COVID‐19 in patients with ankylosing spondylitis (AS) and its relationship with TNF‐a inhibitors

**DOI:** 10.1002/iid3.915

**Published:** 2023-06-14

**Authors:** Shafieh Movassaghi, SeyedAhmad SeyedAlinaghi, Abdolrahman Rostamian, Seyed Reza Najafizadeh, Elham Nezhadseifi

**Affiliations:** ^1^ Rheumatology Research Center, Imam Khomeini Hospital Complex Tehran University of Medical Sciences Tehran Iran; ^2^ Iranian Research Center for HIV/AIDS, Iranian Institute for Reduction of High‐Risk Behaviors Tehran University of Medical Sciences Tehran Iran

**Keywords:** ankylosing spondylitis, biological drugs, COVID‐19, outcomes

## Abstract

**Introduction:**

Ankylosing spondylitis (AS) is a condition that is treated with nonsteroidal anti‐inflammatory drugs and biological drugs such as anti tumor necrosis factor alpha (TNF‐α). This study examined the prevalence of COVID‐19 among individuals with AS and compare it between those receiving and not receiving TNF‐α inhibitors.

**Methods:**

A cross‐sectional study was conducted at the rheumatology clinic of Imam Khomeini Hospital in Tehran, Iran. The study included patients with AS who sought treatment at the clinic. Demographic information, laboratory and radiographic findings, and disease activity were recorded through interviews and examinations using a questionnaire.

**Results:**

A total of 40 patients were studied over the course of 1 year. Among them, 31 patients were administered anti‐TNF‐α drugs, with 15 patients (48.3%) receiving subcutaneous Altebrel (Etanercept), 3 patients (9.6%) receiving intravenous Infliximab, and 13 patients (41.9%) receiving subcutaneous Cinnora (Adalimumab). Of the total, 7 patients (17.5%) tested positive for COVID‐19, 1 of whom was confirmed through both CT scan and polymerase chain reaction (PCR) testing, while the remaining 6 patients were confirmed only through PCR testing. All patients tested positive for COVID‐19 were male, and 6 of them had received Altebrel. Among the 9 AS patients who did not receive TNF inhibitors, 1 patient contracted SARS‐CoV‐2. The clinical symptoms experienced by these patients were mild, and hospitalization was not required. However, 1 patient who had insulin‐dependent type 1 diabetes and was receiving Infliximab required hospitalization. This patient exhibited more severe COVID‐19 symptoms, including high fever, pulmonary involvement, dyspnea, and decreased oxygen saturation. No cases of COVID‐19 were reported in the Cinnora treatment group. The use of any of the drugs did not demonstrate a significant relationship with the occurrence of COVID‐19 in patients.

**Conclusions:**

The use of the TNF‐α inhibitors in patients with AS, may be associated with reduced hospitalization and death rate in COVID‐19 cases.

## Graphical abstract

The use of the biological drugs in patients with ankylosing spondylitis, may be related to reducing hospitalization and death rate of COVID‐19.

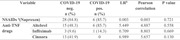

## 1 INTRODUCTION

Ankylosing spondylitis (AS) belongs to a family of diseases known as spondyloarthropathy or spondyloarthritis.^1^ Radiological sacroiliitis is a hallmark of AS.^2^ Inflammation of the sacroiliac joints and spine sometimes leads to bone ankylosis. Spinal ankylosis occurs in the later stages of the disease but does not occur in many patients with mild disease.^3^ Early diagnosis and appropriate treatment of the disease play a critical role in controlling inflammatory symptoms and preventing deformity and limited mobility of the joints and thus preventing disability in patients.^4^

In addition to exercise programs to maintain body composition and range of motion, treatments for AS at the forefront include nonsteroidal anti‐inflammatory drugs (NSAIDs).^5^ Moreover, patients with AS are frequently treated with biologic drugs, which raises concern for infectious complications. Given the pivotal role of tumor necrosis factor‐alpha (TNF‐α) in the development of inflammatory disorders, the administration of TNF‐α inhibitors plays a key role in the treatment of inflammatory diseases.^6^

The outbreak of COVID‐19 caused by the novel SARS‐CoV‐2 led to a large number of infections and deaths worldwide.^7^ Cytokine storm triggered by SARS‐CoV‐2 was documented in several COVID‐19 patients admitted to the intensive care unit, and elevated plasma levels of inflammatory cytokines were associated with disease severity and poor prognosis in these patients.^8^, ^9^

International registries of COVID‐19‐positive patients with inflammatory bowel disease (SECURE‐IBD registry)^10^ or rheumatic diseases (C19‐GRA)^11^ have been developed and analyzed the outcomes of COVID‐19 to record the incidence and prognosis of this infection. In terms of treatments, both registries demonstrated that patients treated with glucocorticoids (GCs) had poor clinical outcomes of COVID‐19, whereas those treated with anti‐TNF therapies, particularly when used as a monotherapy, had a decreased risk of hospitalization due to COVID‐19.^10^, ^11^ These findings suggest that anti‐TNF monotherapy may be protective against severe COVID‐19. Therefore, this study aimed to estimate the prevalence of COVID‐19 disease and its outcomes in AS patients who received three classes of anti‐TNF drugs including Altebrel (Etanercept), Infliximab, and Cinnora (adalimumab).

## 2 MATERIALS AND METHODS

### 2.1 Study population and inclusion/exclusion criteria

In this descriptive‐analytical cross‐sectional study, all patients with AS who were referred to the rheumatology clinic of the Imam Khomeini Hospital in Tehran, Iran, were evaluated by census method for a period of 1 year from February 2020 to 2021.

Inclusion criteria were: (1) definitive diagnosis of AS based on modified New York Criteria^12^ and the Assessment of SpondyloArthritis International Society^13^; (2) Obtaining informed written consent of the patient to participate in the study. The patients were excluded from the study if they did not agree to enter or continue to participate in the project.

### 2.2 Data collection

Patients were first examined by a rheumatologist and their demographic information (age, sex, educational level, marriage status, etc), clinical examination results, and laboratory tests,^14^ Bath AS disease activity index (BASDAI),^15^ as well as medications used including NSAIDs, Infliximab, Altebrel, Cinnora were collected using a questionnaire.

The AS disease activity score (ASDAS) was calculated for patients using the following formula:

ASDAS‐CRP = 0.12 × back pain + 0.06 × duration of morning stiffness + 0.11 × patient global + 0.07 × peripheral pain/swelling + 0.58 × Ln (CRP + 1)

The values less than 1.3 were considered inactive phases, between 1.3 and 2.0 as mild to moderate, between 2.1 and 3.5 as severe, and more than 3.5 as very high disease activity.^16^

In addition, the presence of COVID‐19 infection was confirmed using polymerase chain reaction (PCR) and/or pulmonary CT scan tests.

### 2.3 Statistical analysis

Data analysis was performed using SPSS software (version 25 for Windows; IBM SPSS Statistics) for both descriptive and inferential statistics. The normality was assessed using Kolmogorov−Smirnov *Z*‐test and parametric or nonparametric tests were accordingly selected. Spearman's or Pearson's tests were used to examine the relationship between continuous variables, while the *χ* ^2^ test was used to investigate the relationship between categorical variables. *p* < 0.05 values were considered statistically significant. Also, the likelihood ratio was reported to indicate the value of the Pearson's correlation.

## 3 RESULTS

A total of 40 patients were studied during the 1‐year study period. The mean age of patients was 38.03 ± 10.84 years. The mean duration of the disease was 6.41 ± 4.41 years (range: 1−20). Seven male patients (17.5%) had COVID‐19, of which 6 were positive by PCR test. One patient had more acute symptoms such as severe fever and pulmonary involvement on CT scan due to COVID‐19. Demographic and disease history information of patients are depicted in Table 1.

**Table 1:** Demsographic and disease history information of AS patients divided into positive/negative COVID‐19.

Variable		COVID‐19 negative *n* (%)	COVID‐19 positive *n* (%)	*p* Value
Sex	Male	29 (87.9)	7 (100)	0.582
	Female	4 (12.1)	0	
Age (years old)	≤30	8 (24.2)	2 (28.6)	0.62
	31−49	20 (60.6)	3 (42.8)	
	≥50	5 (15.2)	2 (28.6)	
Educational level	Illiterate	0	1 (14.3)	0.343
	Primary school (years 1−5)	5 (15.2)	1 (14.3)	
	Primary school (years 6−9)	3 (9.1)	0	
	Secondary school (years 10−12)	3 (9.1)	0	
	Secondary school graduate	14 (42.4)	3 (42.9)	
	Bachelor degree	3 (9.1)	1 (14.3)	
	Master degree	5 (15.2)	1 (14.3)	
Marriage status	Married	21 (63.6)	2 (28.6)	0.258
	Single	11 (33.3)	5 (71.4)	
	Divorced	1 (3.0)	0	
Smoking	Negative	20 (60.6)	5 (71.4)	0.751
	Positive	11 (33.3)	2 (28.6)	
	Former	2 (6.1)	0	
Diabetes	Negative	31 (93.9)	6 (85.7)	>0.9
	Positive	2 (6.1)	1 (14.3)	
Hypertension	Negative	26 (78.8)	5 (71.4)	>0.9
	Positive	7 (21.2)	2 (28.6)	

Abbreviation: AS, ankylosing spondylitis.

The mean age (±SD) was 37.7 ± 11.9 and 38.09 ± 10.8 years for COVID‐19 positive and negative groups, respectively. There was no significant association between age and infection rate. The ASDAS, BASDAI, and C‐reactive protein (CRP) values in both groups are presented in Table 2.

**Table 2:** The ASDAS, BASDAI, and CRP values in AS patients with positive/negative COVID‐19 infection.

Variable	COVID‐19 negative mean ± SD	COVID‐19 positive mean ± SD	*p* Value	Disease duration (year)		*p* Value
				≤10	>10	
ASDAS	2.04 ± 1.01	2.40 ± 0.84	0.26	2.32 ± 1.12	2.04 ± 0.96	0.47
BASDAI	2.38 ± 0.99	3.26 ± 1.20	0.20	2.32 ± 0.96	2.57 ± 1.09	0.85
CRP	9.22 ± 11.61	6.17 ± 5.91	0.40	15.0 ± 15.61	7.14 ± 9.01	0.18

Abbreviations: AS, ankylosing spondylitis; ASDAS, AS disease activity score; BASDAI, bath AS disease activity index; CRP, C‐reactive protein.

The mean ASDAS and BASDAI scores in patients who tested positive for COVID‐19 were higher, while CRP values were lower in these patients; however, the differences were not statistically significant. The mean ASDAS and CRP scores were higher in those who were AS patients ≤10 years, while BASDAI score was lower in these patients; also, the differences were not statistically significant. The frequency of use of NSAIDs and anti‐TNF drugs is presented in Table 3.

**Table 3:** The frequency of NSAIDs and anti‐TNF drugs in AS patients with positive/negative COVID‐19 infection.

Variable		COVID‐19 negative *n* (%)	COVID‐19 positive *n* (%)	LR	Pearson's correlation	*p* Value
NSAIDs (Naproxen)		28 (84.8)	6 (85.7)	0.003	0.003	0.721
Anti‐TNF drugs	Altebrel	15 (48.3)	6 (85.7)	5.449	4.887	0.558
	Infliximab	3 (9.6)	1 (14.3)	0.709	8.803	0.669
	Cinnora	13 (41.9)	0	6.989	5.657	0.130

Abbreviations: AS, ankylosing spondylitis; LR, likelihood ratio; NSAID, nonsteroidal anti‐inflammatory drugs; TNF, tumor necrosis factor.

Six patients who tested positive for COVID‐19 were receiving Altebrel medication. The clinical symptoms experienced by these individuals, including cough and myalgia, were so mild that hospitalization was not required for any of them. However, 1 patient who was receiving Infliximab and had type 1 diabetes, requiring insulin treatment, needed to be hospitalized. This particular patient exhibited more severe COVID‐19 symptoms, such as high fever and pulmonary involvement observed on a CT scan, dyspnea, and decreased oxygen saturation. Additionally, no cases of COVID‐19 were reported in the Cinnora treatment group. The use of anti‐inflammatory and anti‐TNF drugs did not show a significant correlation with COVID‐19.

## 4 DISCUSSION

### 4.1 Anti‐TNF therapy and effect on COVID‐19 outcomes in autoimmune diseases

The treatment with anti‐TNF‐α agents is beneficial for patients with AS by improving pain, function, and disability.^17^ High levels of TNF have been reported in COVID‐19 patients, and an early increase in TNF is a predictor of higher mortality in these patients.^18^ Besides, induced cytokine storm by COVID‐19 infection involves raising TNF level, which has proinflammatory activities that can lead to extensive tissue damage, including pulmonary injury and shock through vascular leakage.^19^, ^20^ The documented evidence revealed that anti‐TNF biological agents could be one potential treatment that deserves higher priority in COVID‐19 trials.^21^ Administration of anti‐TNF to patients for the treatment of autoimmune disease leads to reductions in all of these key inflammatory cytokines.^21^

Anti‐TNF therapy has shown an inverse association with the outcome of death or hospital admission in COVID‐19 patients with inflammatory bowel diseases.^22^ However, the use of anti‐TNF therapy did not have an impact on intensive care admission, ventilation, or death in COVID‐19 patients.^22^ Several studies have reported significant improvements in COVID‐19 symptoms, chest imaging, inflammatory markers, and cytokine concentrations in patients with inflammatory bowel disease and concomitant COVID‐19 infection who received infliximab treatment.^23^, ^24^

Monotherapy of anti‐TNF in rheumatic disease has been associated with a lower rate of hospital admission for COVID‐19 patients.^25^ Another survey revealed that among rheumatic patients with COVID‐19 who were treated with immunomodulatory drugs, 48% of patients required ventilator support and 12% succumbed to the disease. However, none of the patients receiving anti‐TNF therapy required ventilator support or died. Notably, 40% of patients on non‐TNF biologics required ventilator support, and 13% died, underscoring the importance of specific TNF blockade.^26^ Patients with rheumatological diseases and AS that used immunosuppressive drugs, including anti‐TNF therapy, are considered to potentially be at a higher risk for infections and their complications.^27^

Administration of anti‐TNF has been observed to result in the reduction of acute‐phase proteins, including CRP, serum amyloid A, haptoglobin, and fibrinogen, which are elevated in patients with rheumatoid arthritis (RA).^28^ Consistent with these findings, the present study revealed that COVID‐19‐positive patients had lower mean levels of CRP compared to other patients. Furthermore, the use of anti‐TNF drugs has been associated with a connection between clotting and a notable decrease in d‐dimer and pro‐thrombin fragments within 1 h of drug administration.^29^ Therefore, anti‐TNF may also mitigate COVID‐19‐induced thrombosis.

### 4.2 Other cytokine blockers and their effect on COVID‐19 outcomes

The first randomized controlled phase 3 trial of tocilizumab, an IL‐6 antagonist, in COVID‐19 infection did not show any difference in clinical status or mortality.^30^ Similarly, an exploratory phase 2 trial of sarilumab also did not demonstrate any improvement in clinical outcomes.^31^ However, data from a recent meta‐analysis concerning all‐cause mortality in critically ill patients indicated lower rates at 28 days after randomization for both tocilizumab and sarilumab compared with placebo or usual care.^32^ Moreover, in RA patients, receiving treatment with anti‐TNF therapy for the underlying rheumatic disease was significantly associated with lower odds of COVID‐19‐related hospitalization, in contrast to not receiving any anti‐TNF inhibitor therapy.^33^

### 4.3 Other immunosuppressive therapies and COVID‐19 outcomes

Studies in patients infected with coronavirus and influenza virus treated with corticosteroids showed a higher risk of complications and death.^34^ Poor clinical outcomes of GCs treatment in COVID‐19 patients were observed in both patients with rheumatic diseases (C19‐GRA3 registry)^25^ and inflammatory bowel disease (SECURE‐IBD registry).^22^ Similarly, another study by Cooksey et al.,^35^ demonstrated an increased risk of death associated with GCs use in the registry of inflammatory arthritis patients in the United Kingdom.

## 5 CONCLUSION

The use of anti‐TNF drugs in AS patients who were infected with COVID‐19 was associated with a mild form of infection, without the need for hospitalization. However, no statistically significant correlation was found between anti‐TNF therapy and the severity or outcomes of COVID‐19. Due to the small sample size, the results of this study should not be generalized to all AS patients with COVID‐19 infection. Although the modest effect observed with these agents is promising, further studies should be conducted to explore the efficacy of other agents with higher efficiency. The potential of anti‐TNF therapy as a treatment for COVID‐19 is supported by both biological plausibility and observational clinical data. Further multicenter studies are needed to investigate this hypothesis.

## AUTHOR CONTRIBUTIONS

**Shafieh Movassaghi and Elham Nezhadseifi**: Conceptualization, writing—original draft. **SeyedAhmad SeyedAlinaghi and Abdolrahman Rostamian**: Analyzed the data, and reviewed and edited it. **Seyed Reza Nejafizadeh**: Contributed to the questionnaire, review, and editing. All the authors have read and approved the final manuscript.

## CONFLICT OF INTEREST STATEMENT

The authors declare no conflict of interest.

## Data Availability

The data supporting the study are available via the authors.
